# The Non-Gastric H^+^/K^+^ ATPase (ATP12A) Is Expressed in Mammalian Spermatozoa

**DOI:** 10.3390/ijms23031048

**Published:** 2022-01-19

**Authors:** Maria Favia, Andrea Gerbino, Elisabetta Notario, Vincenzo Tragni, Maria Noemi Sgobba, Maria Elena Dell’Aquila, Ciro Leonardo Pierri, Lorenzo Guerra, Elena Ciani

**Affiliations:** Department of Biosciences, Biotechnologies and Biopharmaceutics, University of Bari “Aldo Moro”, Via E. Orabona 4, 70125 Bari, Italy; mariafavia@hotmail.com (M.F.); andrea.gerbino@uniba.it (A.G.); e.notario@studenti.uniba.it (E.N.); Vincenzo.tragni@uniba.it (V.T.); marianoemi.sgobba@gmail.com (M.N.S.); mariaelena.dellaquila@uniba.it (M.E.D.); ciro.pierri@uniba.it (C.L.P.); elena.ciani@uniba.it (E.C.)

**Keywords:** H^+^/K^+^ ATPase, spermatozoa, *Bos taurus*, *Bubalus bubalis*, *Ovis aries*, Western blot, immunofluorescence, in silico sequence analysis, 3D molecular modelling

## Abstract

H^+^/K^+^ ATPase Type 2 is an heteromeric membrane protein involved in cation transmembrane transport and consists of two subunits: a specific α subunit (ATP12A) and a non-specific β subunit. The aim of this study was to demonstrate the presence and establish the localization of ATP12A in spermatozoa from *Bubalus bubalis*, *Bos taurus* and *Ovis aries*. Immunoblotting revealed, in all three species, a major band (100 kDa) corresponding to the expected molecular mass. The ATP12A immunolocalization pattern showed, consistently in the three species, a strong signal at the acrosome. These results, described here for the first time in spermatozoa, are consistent with those observed for the β_1_ subunit of Na^+^/K^+^ ATPase, suggesting that the latter may assemble with the α subunit to produce a functional ATP12A dimer in sperm cells. The above scenario appeared to be nicely supported by 3D comparative modeling and interaction energy calculations. The expression of ATP12A during different stages of bovine sperm maturation progressively increased, moving from epididymis to deferent ducts. Based on overall results, we hypothesize that ATP12A may play a role in acrosome reactions. Further studies will be required in order to address the functional role of this target protein in sperm physiology.

## 1. Introduction

The “non gastric” H^+^/K^+^ ATPase is a transmembrane heterodimer responsible for H^+^ efflux in exchange with K^+^. It consists of an α and a β subunit [1,2] for which their assembly is required for their functional expressions. The α subunit specific for the “non gastric” H^+^/K^+^ ATPase is also known as ATP12A. However, the identity of the β subunit interacting with ATP12A is still controversial [3]. From studies carried out in various cell types, the β subunit of the “gastric” H^+^/K^+^ ATPase (i.e., ATP4B) and one of the β subunits of the Na^+^/K^+^ ATPase, known as ATP1B1, ATP1B2 and ATP1B3, have been proposed as possible candidates for interaction with ATP12A [1,4,5,6,7,8,9]. Together with the “gastric” H^+^/K^+^ ATPase and Na^+^/K^+^ ATPase, the “non gastric” H^+^/K^+^ ATPase forms the subgroup of the so-called X-K-ATPases, which is member of the P2C-type ATPases, a large family of integral membrane transporters responsible for generating and maintaining essential electro-chemical gradients across cellular membranes by pumping ions using the energy from ATP hydrolysis [10,11]. X-K-ATPases transporters are conserved across many species, and they can be observed ubiquitously in all cell types (as in the case of Na^+^/K^+^ ATPase) or with a less broad patterns of expression. Notably, the “non-gastric” H^+^/K^+^ ATPase protein has been described in several areas, including colon [12], kidney [13], prostate [4,5], human placental syncytiotrophoblast [14], tracheal and airway epithelia [15,16], neutrophils [17,18,19], Madin-Darby Canine Kidney (MDCK) cells [7], pancreatic duct cell lines [20,21], gastric cancer cells [22], pro-myelocytic cells [23] and insulinoma cells [24], and is overexpressed in colorectal carcinoma [25] and prostate cancer cells [26]. Proteomic studies also detected “non-gastric” H^+^/K^+^ ATPase in mice brain, heart, kidney, lung, pancreas, brown adipose tissue, spleen, testis [27], liver [28] and in human sperm cells [29]. For each X-K-ATPases transporter, protein complexes and their relative subunits are indicated in Table 1.

Here, we investigated for the first time ATP12A protein expression and localization in frozen/thawed spermatozoa from cattle, sheep and buffaloes. To check for possible localization pattern overlapping, we also investigated, in the three mentioned species, the pattern of protein localization of the Na^+^/K^+^ ATPase isoforms most represented in sperm cells, namely, ATP1A1 and ATP1A4 for the α subunit [30,31,32,33,34,35,36,37,38,39,40,41,42,43,44] and ATP1B1 for the β subunit [36,45,46]. Moreover, for bovine species, ATP12A protein localization was investigated in spermatozoa collected from epididymis and deferent ducts in order to highlight possible pattern changes during sperm maturation. To confirm the specificity of the adopted antibodies as well as to provide support to the hypothesis of a possibly functional interaction between ATP12A and ATP1B1 formulated in light of our experimental results, a thorough in silico analysis was also performed.

## 2. Results

### 2.1. ATP12A Shows Conserved Sequence Features Detectable along Phylogenetically Distant Species That Determine a Dedicated Cluster in Phylogenetic Analysis When Compared to Other P2C-ATPase Family Members

**Figure 1:**
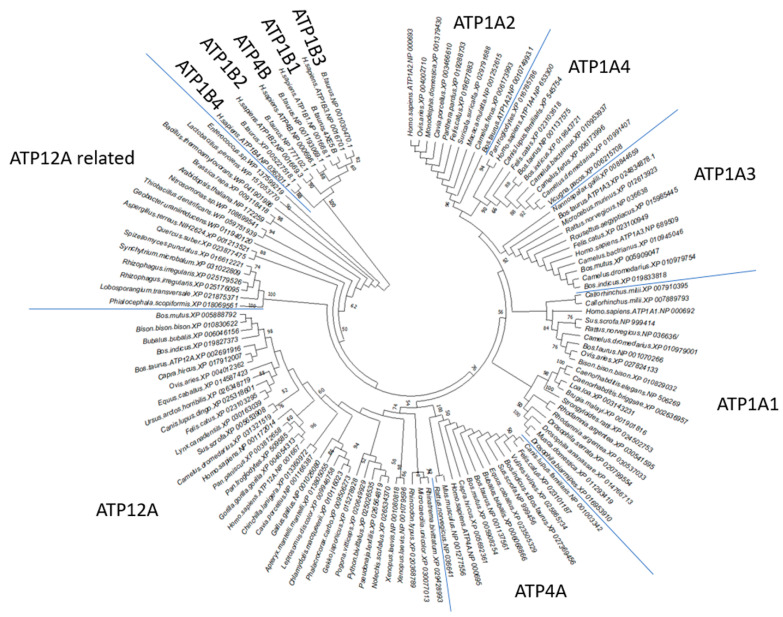
Phylogenetic tree of P2C-ATPase homologous sequences. Maximum likelihood phylogenetic tree of P2C-ATPase homologous protein sequences selected from representative species of metazoan, nematodes, fungi, bacteria and plants. Each tree leaf reports the corresponding organism and *RefSeq* protein accession number. Nodes supported by bootstrap values are indicated by labels.

P2C-ATPase sequences are clearly detectable in all metazoans. Notably, in *Mammalia*, it is possible to observe a specialization of P2C-ATPase sequences that reflect tissue specific needs [11]. By using the *B. taurus* ATP12A sequence as a query sequence for screening other taxonomic groups, based on the above-described thresholds of E-value, percentage of identical residues and query coverage, it was possible to sample 36 sequences from metazoan and 7, 3 and 6 sequences with high similarity to *B. taurus* ATP12A also from more phylogenetically distant fungi, plants and bacteria, respectively. Due to the high sequence similarity demonstrated by P2C-ATPase members, ATP1A1-4 and ATP4A homologous sequences were sampled and aligned to ATP12A and used in the provided phylogenetic analyses, confirming that their sequence features reflect their abilities in grouping in dedicated clusters of sequences (Figure 1).

### 2.2. Multiple Sequence Alignment (MSA) Highlights ATP12A Specific Sequence Features in the Targeted Epitopes despite the High Percentage of Identical Amino Acids Shared with Other α Subunits of P2C-ATPases Members

Among the 122 ATP12A sampled homologous sequences, an extract of 42 homologous metazoan P2C-ATPases, with specific reference to ATP12A (11 sequences), ATP1A1 (6), ATP1A2 (5), ATP1A3 (5), ATP1A4 (6) and ATP4A (9), was selected for the following comparative analyses. Notably, all 42 analyzed sequences show a high percentage of identical residues among different P2C-ATPases ranging between 57 and 88% (Table S1).

The percentage of identical residues between orthologs (i.e., members of the only ATP12A or ATP1A1 and so on) ranges between 85% and 98%. *Bos taurus* ATP12A and its counterparts (orthologs) from *Ovis aries* and *Bubalus bubalis* full length sequences share ATP1A1, ATP1A2, ATP1A3, ATP1A4 and ATP4A paralogs with 60–64% of identical residues (see Table S1), with higher differences in correspondence of the N-terminal region and extra-membrane loops.

Given the lack of commercial antibodies claimed to react against bovine, bubaline or ovine ATP12A, we explored the identity of residues between immunogens and protein sequences from the above species. The anti-ATP12A A62134 EpiGentek antibody had been obtained by using human 171-306 protein residues (NP_001667.4) as immunogenic peptide, which resulted in sharing 85%, 84% and 83% identical residues, respectively with *Bos taurus*, *Bubalus bubalis* and *Ovis aries* ortholog-corresponding regions (XP_002691916.3; XP_006046156; XP_004012362), hence justifying its use in the considered species [47,48]. Conversely, the human ATP12A 171-306 immunogenic sequence shares, with their aligned counterparts in ATP4A, ATP1A4, ATP1A2, ATP1A1 and ATP1A3 retrievable from *B. taurus, B. bubalis* and *O. aries*, 73%, 70%, 69%, 67% and 65% of identical residues, respectively, thus pointing to antibody specificity towards ATP12A (Figures S1 and S2).

### 2.3. MSA Highlights ATP1B1-Specific Sequence Features in the Targeted Epitopes and a Low Percentage of Identical Amino Acids Shared with Others β Subunits of P2C-ATPases Members

The full-length ATP1B1-4 and ATP4B homologous sequences show a percentage of identical residues highly variable for each compared subunit subgroups. Indeed, the percentage of identical residues intra-subunit subgroup (i.e., between orthologs) ranges between 80% and 99%, whereas the percentage of identical residues inter-subunit subgroup (i.e., between paralogs) ranges between 28% and 40% (Table S2). The human ATP1B1 64-241 immunogenic sequence shares 29% of identical residues with ATP4B and approximately 31–33% of identical residues with *Bos taurus* ATP1B2, ATP1B3 and ATP1B4. Thus, it appears that the cited antibody is selective versus ATP1B1 (see Figures S3 and S4). The above human 64-241 ATP1B1 amino acid region shares 87% of identical residues with *Bos taurus, Ovis aries* and *Bubalus bubalis* ortholog-corresponding regions (4xe5_b.pdb.fa, NP_001009796.1 and XP_006054394.1, respectively).

### 2.4. MSA Highlights ATP1A1 and ATP1A4 Specific Sequence Features in Targeted Epitopes and a Low Percentage of Identical Amino Acids Shared with Other α Subunits of P2C-ATPases Members

It is a commonly held view that cross-reactivity requires more than 70% sequence identity, while proteins that share <50% sequence identity are rarely cross-reactive [47,48]. The ovine anti-ATP1A1 Invitrogen antibody is reported to be active against the 496- EQPLDEELKDA-506 protein peptide that resulted in sharing 100% identical residues with the corresponding amino acid fragments in *Bos taurus* and *Bubalus bubalis* (see Supplementary Figure S5). Therefore, we are highly confident that the monoclonal ATP1A1 antibody recognizes the bovine (and the bubaline) proteins. In addition, it shares 62% and 54% of identical residues with ATP1A4 and ATP12A, respectively, in *B. taurus* and *B. bubalis* corresponding protein regions; hence, based on the above, we consider ovine anti-ATP1A1 antibody as having higher probabilities of detecting a specific signal rather than aspecific ones. The human anti-ATP1A4 E-AB-65236 Elabscience antibody is reported to be active against the 1-90 protein peptide (Q13733), which resulted in sharing 64%, 62% and 65% of identical residues, respectively, with the *Bos taurus, Bubalus bubalis* and *Ovis aries* ortholog-corresponding regions (see Figure S5). Despite the fact that these values are lower than the above-mentioned 70% threshold, we still could detect positive staining in our study at the flagellum in all three considered species (see Section 2.6), which we consider having a higher probability of representing specific signals rather than aspecific ones. Indeed, the human anti-ATP1A4 E-AB-65236 Elabscience antibody shares generally lower percentages of identical residues with *Bos taurus, Bubalus bubalis* and *Ovis aries* ATPA1 (43%, 44% and 61%, respectively) and ATP12A (40%, 39% and 39%, respectively). Moreover, the only AA stretch shared by the compared proteins concerns residues 57-DDHKL-61, which is again poorly exposed, beyond its small size.

### 2.5. ATP12A Is Expressed in Frozen/Thawed Sperm Cells

**Figure 2:**
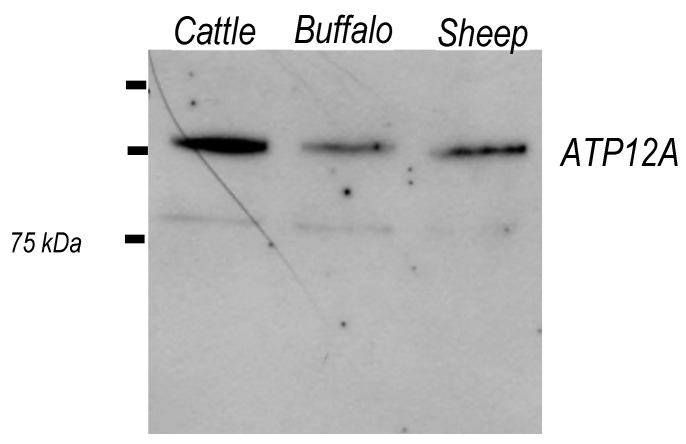
Western blot of sperm homogenates from cattle, buffalo and sheep samples. Immunocomplexes obtained using the EpiGentek A62134 antibody against ATP12A and an anti-rabbit secondary antibody (Merck/MilliporeSigma) conjugated to peroxidase were detected at about 100 kDa using the ECL plus reagent (GE Healthcare Life Sciences; see Materials and Methods for details).

Western blot analysis of frozen/thawed sperm homogenates from the species *Bos taurus*, *Bubalus bubalis* and *Ovis aries* was performed by using a commercial antibody (EpiGentek) against ATP12A. As shown in Figure 2, a major band was observed for all species at about 100 kDa, corresponding to the expected molecular weight based on the literature for different cell lines [3,20,24,26,49,50,51,52]. A faint and possibly unspecific band at 75 kDa was also observed. Based on the published literature [32,35,44], a molecular mass of about 100 kDa on electrophoretic gel is also reported for ATP1A1-4 isoforms of the Na^+^/K^+^-ATPase.

### 2.6. ATP12A Localizes at the Acrosomal Region in Frozen/Thawed Sperm Cells

**Figure 3:**
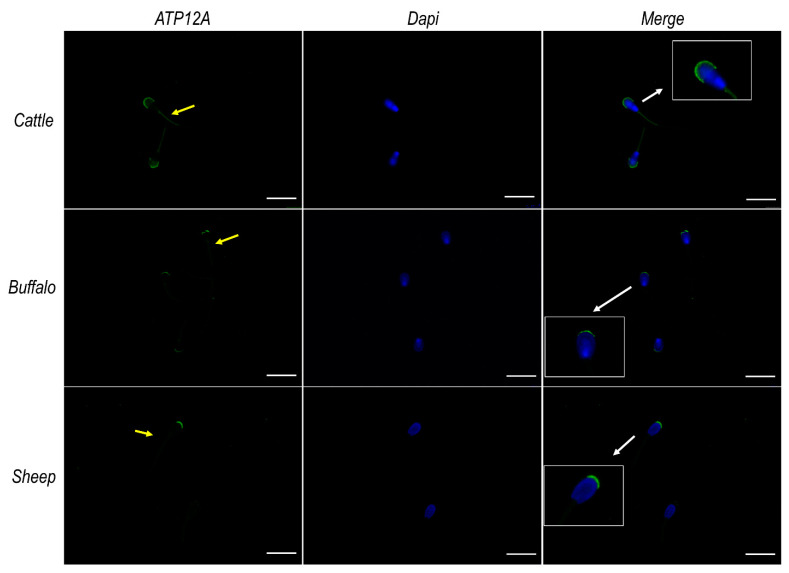
ATP12A protein expression in sperm cells from cattle, buffalo and sheep. Representative fluorescence microscope images showing immunodetection of ATP12A (green) in frozen/thawed sperm cells. The secondary antibody was conjugated to Alexa Fluor 488. Nuclei were counterstained with DAPI (blue). Insets in the merge panels represent 2× magnification of representative sperm cells indicated by white arrows. Yellow arrows within the green channel indicate positive staining of ATP12A at the flagellum. Scale bar: 20 μm.

**Figure 4:**
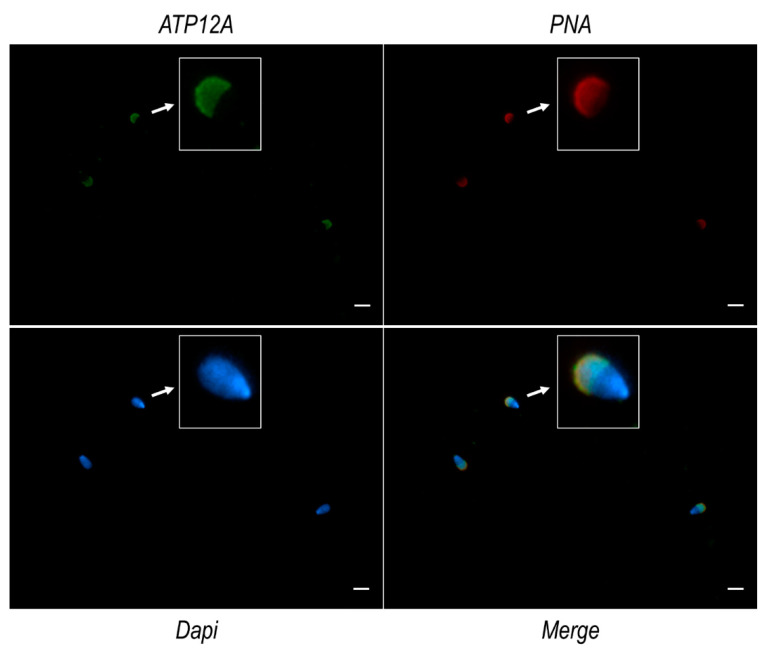
ATP12A and PNA localization in sperm cells from cattle. Representative fluorescence microscope images showing immunodetection of ATP12A (green) and TRITC-conjugated PNA (red) in frozen/thawed sperm cells. The secondary antibody for ATP12A was conjugated to Alexa Fluor 488. Nuclei were counterstained with DAPI (blue). Insets in the four panels represent 4× magnification of the same representative sperm cell as indicated by the white arrows. Scale bar: 10 μm.

Immunofluorescence analysis on frozen/thawed spermatozoa from cattle, buffalo and sheep (Figure 3), performed using the antibody mentioned in the section above, consistently revealed a marked signal at the acrosomal region of the sperm head and a weaker signal at the flagellum middle piece. Negative controls are provided in Supplementary Figure S6. The acrosomal localization of ATP12A was also confirmed by co-staining experiments performed using TRITC-conjugated PNA (Figure 4), which is known to bind to the outer acrosomal membrane [53].

### 2.7. Na^+^/K^+^ ATPase Subunits α_1_ and α_4_ Do Not Colocalize with ATP12A at the Acrosomal Region in Frozen/Thawed Sperm Cells

**Figure 5:**
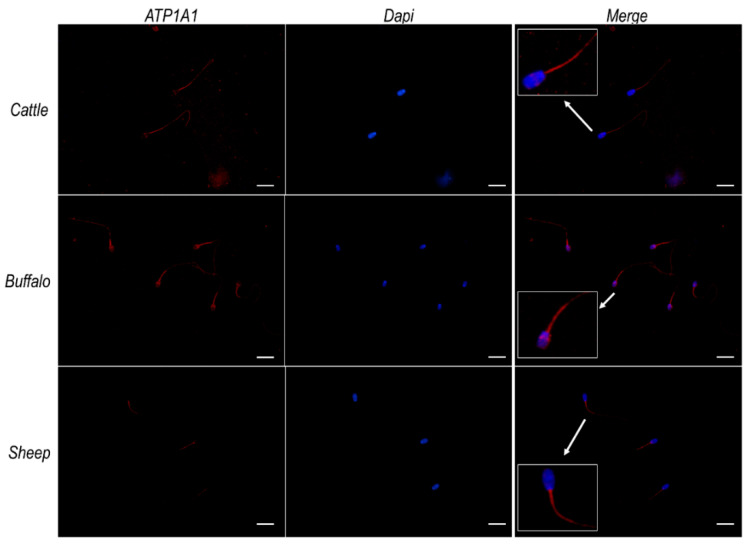
ATP1A1 protein expression in sperm cells from cattle, buffalo and sheep. Representative fluorescence microscope images showing immunodetection of ATP1A1 (red) in frozen/thawed sperm cells. The secondary antibody was conjugated to Alexa Fluor 568. Nuclei were counterstained with DAPI (blue). Insets in the merge panels represent 3× magnification of representative sperm cells indicated by white arrows. Scale bar: 20 μm.

**Figure 6:**
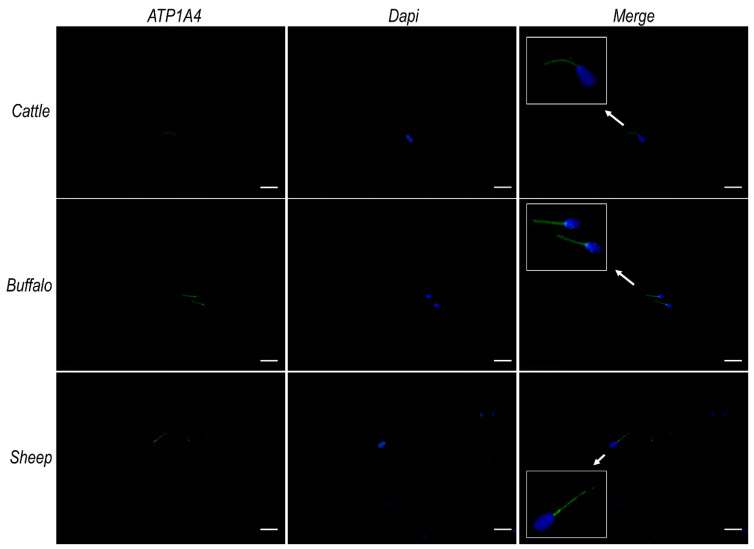
ATP1A4 protein expression in sperm cells from cattle, buffalo and sheep. Representative fluorescence microscope images showing immunodetection of ATP1A4 (green) in frozen/thawed sperm cells. The secondary antibody was conjugated to Alexa Fluor 488. Nuclei were counterstained with DAPI (blue). Insets in the merge panels represent 3× magnification of representative sperm cells indicated by white arrows. Scale bar: 20 μm.

Given the similarity known from the literature [1,54] as well as from our in silico analysis (Table S1) among ATP12A and the two α subunits (α_1_ and α_4_) of Na^+^/K^+^ ATPase previously observed in sperm cells [30,31,32,33,34,35,36,37,38,39,40,41,42,43,44], we repeated immunofluorescence analysis using, respectively, a monoclonal antibody raised against ATP1A1 (Invitrogen) and a polyclonal antibody raised against ATP1A4 (Elabscience) of the Na^+^/K^+^ ATPase. The observed results (Figure 5 and Figure 6, respectively) that consistently pointed to a strong signal at the sub-equatorial region of the sperm head and the flagellum middle piece, coupled to a weaker signal over the remaining portion of the flagellum. On the contrary, no signals were detected in the considered species at the acrosomal region, where we had previously observed ATP12A (Figure 3). Negative controls are provided in Supplementary Figure S6.

### 2.8. ATP12A Colocalizes with Na^+^/K^+^ ATPase Subunit β_1_ at the Acrosomal Region in Frozen/Thawed Sperm Cells

**Figure 7:**
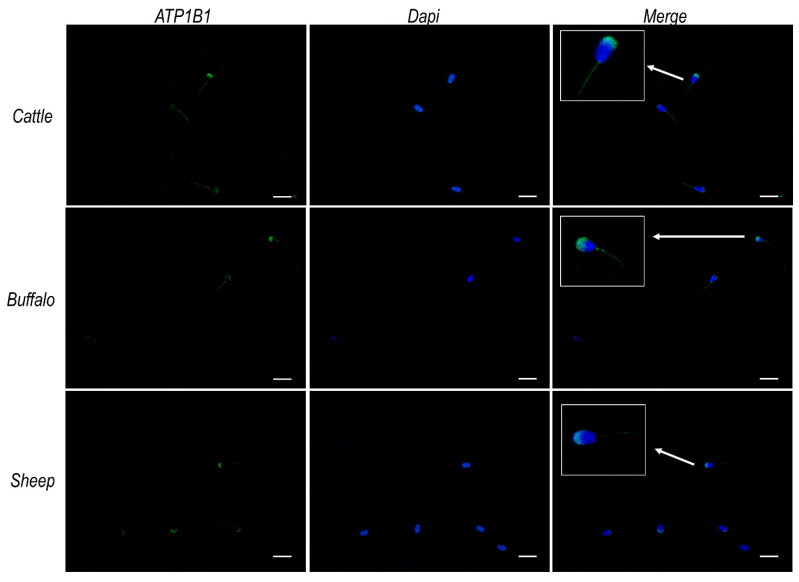
ATP1B1 protein expression in sperm cells from cattle, buffalo and sheep. Representative fluorescence microscope images showing immunodetection of ATP1B1 (green) in frozen/thawed sperm cells. The secondary antibody was conjugated to Alexa Fluor 488. Nuclei were counterstained with DAPI (blue). Insets in the merge panels represent 3× magnification of representative sperm cells indicated by white arrows. Scale bar: 20 μm.

Immunofluorescence analysis performed using an antibody raised against the β_1_ subunit of Na^+^/K^+^ ATPase (Cusabio) evidenced, for all considered species, an intense signal at the acrosomal region of the sperm head and at the flagellum’s middle piece, as well as a weaker signal over the remaining portion of the flagellum (Figure 7).

### 2.9. Expression of ATP12A Changes during Sperm Maturation

**Figure 8:**
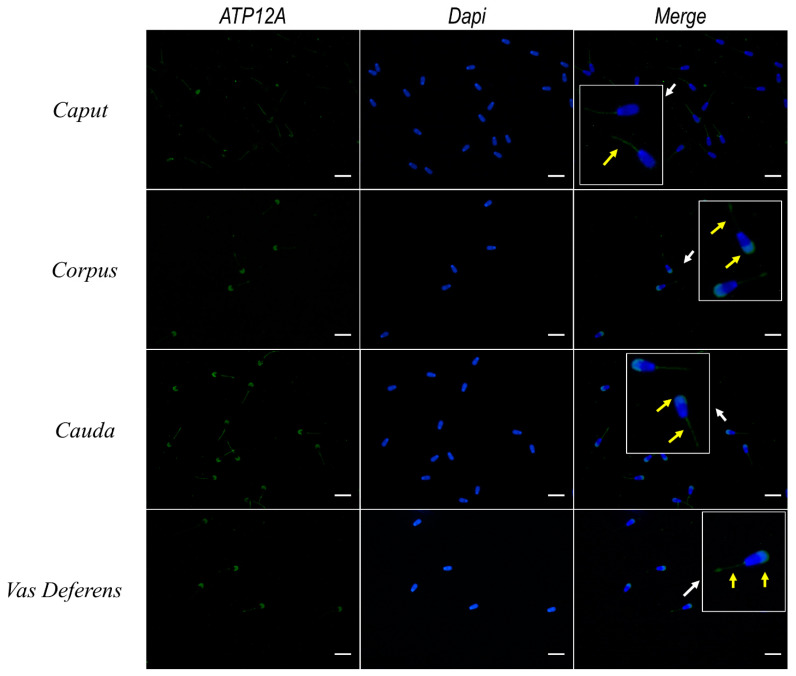
Immunofluorescence of ATP12A in fresh bovine spermatozoa from *caput*, *corpus* and *cauda* epididymis and from the *vas deferens*. Representative fluorescence microscope images showing immunodetection of ATP12A (green). The secondary antibody was conjugated to Alexa Fluor 488. Nuclei were counterstained with DAPI (blue). Insets in the merged panels represent 3× magnification of representative sperm cells indicated by white arrows. Yellow arrows within insets indicate positive staining of ATP12A. Scale bar: 20 μm.

Sperm cells collected from testis, different sections of epididymis (*caput*, *corpus* and *cauda*) and deferent ducts were subjected to immunofluorescence using the set of antibodies previously adopted for analysis of frozen/thawed spermatozoa. As shown in Figure 8, the majority of sperm cells from caput epididymis presented a weak signal for ATP12A at the flagellum’s middle piece, while only a fraction of them presented the classical signal at the acrosomal region previously observed in frozen/thawed spermatozoa. On the other side, all spermatozoa obtained from the remaining portions of epididymis and deferent ducts presented a clear signal at the acrosomal region, together with a weaker signal at the flagellum’s middle piece.

### 2.10. 3D Comparative Modeling of the Bovine ATP12A.ATP1B1 and ATP12A.ATP4B

**Figure 9:**
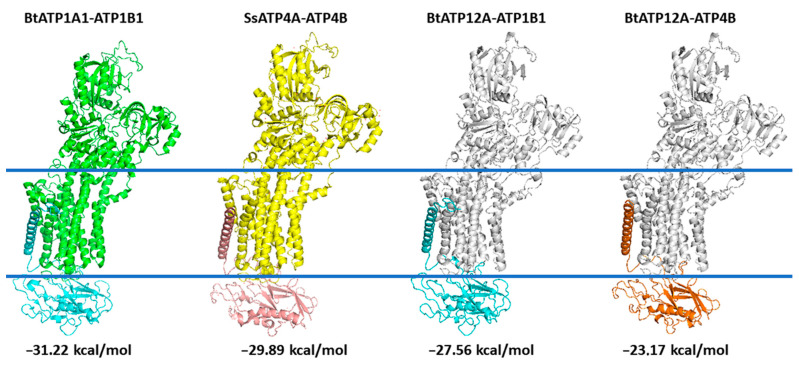
The lateral views of 3D crystallized structures of bovine ATP1A1 in complex with ATP1B1 and of swine ATP4A in complex with ATP4B are reported in green/cyan and yellow/pink cartoon representation, respectively. The 3D comparative models of the bovine ATP12A (white cartoon) in complex with ATP1B1 (cyan cartoon) or ATP4B (orange cartoon) are also reported in cartoon representation. calculated interaction energies are reported.

The protein complex ATP12A.ATP1B1, obtained as described in the Materials and Methods section after relaxation on the *yasara* minimization server, showed a root-mean-square deviation (RMSD) lower than 1.06 Å with crystallized bovine ATP1A1.ATP1B1, whereas it showed an RMSD lower than 2.09 Å with the crystallized swine ATP4A.ATP4B protein complex (Figure 9).

Similarly, protein complex ATP12A.ATP4B showed an RMSD lower than 2.33 Å with crystallized bovine ATP1A1.ATP1B1 (4xe5.pdb), whereas it showed an RMSD lower than 1.47 Å with the crystallized swine ATP4A.ATP4B (5ylu.pdb) protein complex (Figure 9). Notably, the RMSD between coordinates of the two crystallized structures, 4xe5.pdb (ATP1A1.ATP1B1) and 5ylu.pdb (ATP4A.ATP4B), was close to 1.79 Å.

### 2.11. Interaction Energy Calculation

The interaction energies calculated between subunits α and β of the crystallized protein complex and between subunits α and β of the two 3D models always produced a negative value (Table S3, Figure 9), confirming that there might be a binding interaction between ATP12A and ATP1B1/ATP4B. Similar interaction energies obtained for crystallized ATP1A1.ATP1B1 (−31.22 Kcal/mol) and ATP4A.ATP4B (−29.89 Kcal/mol) and for ATP12A.ATP1B1 (−27.56 Kcal/mol) and ATP12A.ATP4B (−23.17 Kcal/mol) 3D comparative models (Table S3, Figure 9) indirectly validated the results obtained for the 3D models. Notably, the interaction energy calculated for the ATP12A.ATP4B complex was the weakest (−23.17 Kcal/mol) among those investigated.

## 3. Discussion

P-type adenosine triphosphatase (ATPases) constitutes a large family of evolutionary related integral membrane ionic transporters that are of vital importance in all kingdoms of life. The electrochemical gradients created and maintained by P-type ATPases are essential as they are involved in a number of vital processes such as secretion and absorption of solutes, control of cell volume, ionic homeostasis and pH [55]. In sperm cells, evidence has been provided concerning (i) the presence of the Na^+^/K^+^ ATPase protein complex, notably, as for the α subunits, both α_1_ and α_4_ [35,40,56,57,58,59,60]; (ii) its cellular localization, consistently reported to pertain to the flagellum (particularly, the mid-piece portion) and only rarely and scarcely detected in the sperm head [32,35,36,37,40,44,61] with the exclusion of the studies performed by [31,41,46] where multiple isoforms of the Na^+^/K^+^ ATPase subunits are reported to localize at the sperm head and not at the flagellum in the bovine species; and (iii) its functional role [36,38,57,61,62]. Notably, in sperm cells, Na^+^/K^+^-ATPase (mainly the ATP1A4 isoform) has been shown to play an important role in cell homeostasis (intracellular Na^+^ and Ca^++^ concentration and pH), motility, hyperactivation and flagellar shape [32,36,38,42,43,44,57,61,62]. These results indicate that Na^+^/K^+^ 1A4 is the main contributor to sperm motility. A more limited number of studies have pointed out the presence of H^+^/K^+^ ATPase Type 2 (alias ATP12A) at both the transcriptome [63,64] and the protein [29] level in mammalian (human, mouse) and avian (chicken) sperm cells. In a recent paper, Escoffier J et al. [65] speculated that, in addition to Na^+^/H^+^ exchangers (NHE), HCO_3_^−^ transporter and HV1 channels, “non gastric” H^+^/K^+^ ATPase could also contribute to the alkalization of the cytoplasmic compartment occurring during capacitation, which is a key step in sperm physiology. Our main goal in the present study was, hence, to confirm the presence of ATP12A in sperm cells from ruminant species (*Bos taurus*, *Bubalus bubalis* and *Ovis aries*); to define its cellular localization in the three considered species; and to check for the presence, in bovine species, of patterns of ATP12A expression during sperm maturation.

We first confirmed the presence of ATP12A in sperm cells via Western blot analysis, thus complementing the reported occurrence by Wang et al. [29] based on mass spectrometry analysis. Due to the lack of a commercial antibody raised against ATP12A in ruminant species, we adopted in this study an anti-human ATP12A antibody for which its specificity was strongly supported by thorough in silico analysis. The Western blot analysis produced, in all three considered species, a single band corresponding to the molecular mass previously reported in other cell types from different species, thus corroborating antibody specificity.

Furthermore, this study addresses for the first time the cellular localization of ATP12A in sperm cells. Consistently across species, as well as across different segments of the reproductive tract, the protein (whenever present) was predominantly observed at the acrosomal region of the sperm head. Interestingly, our immunofluorescent experiments highlighted a pattern of localization coinciding to the one observed for ATP12A and also for the β_1_ subunit of Na^+^/K^+^ ATPase but not for the α_1_ and α_4_ subunits of the αβ Na^+^/K^+^ ATPase heterodimer, which are known to be the most represented Na^+^/K^+^ ATPase α isoforms in sperm cells. Analogously to what has been performed for ATP12A, we were compelled to adopt an anti-human antibody also for ATP1B1 [8], with a preliminary in silico analysis strongly supporting the specificity toward the target protein in the three considered ruminant species (87% of identical residues vs. 29–33% of identical residues with the *Bos taurus* ATP4B, ATP1B2, ATP1B3 and ATP1B4).

Previous evidence from cell types other than spermatozoa has highlighted the functional interaction between the β_1_ subunit of Na^+^/K^+^ ATPase and ATP12A [3,4,5,8]. The β_1_ subunit of the colonic H^+^/K^+^-ATPase assembles with Na^+^/K^+^-ATPase in kidney and distal colon [8,9,66]. Moreover, in an in vitro co-expression study, Swarts et al. [51] highlighted considerable ATPase activity only when ATP12A was coupled with the β_1_ subunit of Na^+^/K^+^ ATPase, while activity was 50-times lower when coupled with the β-subunit of the “gastric” H^+^/K^+^ ATPase, and no activity was detected when coupled with the β_3_ subunit of the Na^+^/K^+^ ATPase. Based on the above, we formulate a hypothesis, corroborated by our interaction energy calculations, stating that a possible functional interaction may exist between ATP12A and the β_1_ subunit of Na^+^/K^+^ ATPase also in sperm cells.

We further detected a clear tendency to a more marked expression of ATP12A moving from epididymis toward deferent ducts. These results seems to be in line with the literature evidencing that immature testicular spermatozoa undergo many maturational changes during epididymal transit in order to acquire the ability to successfully fertilize the oocyte [67]. One of the essential steps in mammalian fertilization is represented by the acrosome reaction, a complex exocytic event. Literature evidence, as well as a mathematical model for the human sperm acrosome reaction, supports the hypothesis that an increase in intra-acrosomal pH can trigger an acrosome reaction [68]. A putative Na^+^/H^+^ exchanger in the acrosome membrane has been proposed to participate in the H^+^ outward current [69,70]; however, its existence has not been established. Based on the above and on the prominently acrosomal cellular localization of ATP12A observed in our study, we are prompted to speculate that ATP12A may contribute in the acrosomal H^+^ outward current needed for triggering the acrosome reaction. Moreover, before the sperm undergoes acrosome reaction, ATP12A could play a role in maintaining the intra-acrosomal pH around 5.3 [71,72] by counteracting the acrosomal V-ATPase-mediated acidification. Since the acrosome is a large lysosome-like vesicle and based on the very recent evidence by Lee and Koh [73] that gastric H^+^/K^+^ -ATPase is expressed in the lysosomes of mice astrocytes where it has been shown to represent an alternative cAMP/PKA-mediated route of proton entry for lysosomal acidification in case of V-ATPase dysfunction, we cannot a priori exclude that a similar feature may also apply to non-gastric ATP12A isoforms detected in our study. The lack of inhibitors specific for ATP12A currently represents a limiting factor for testing the above hypothesis concerning a role of ATP12A in sperm physiology. However, new technologies based on virtually screening chemical libraries [74,75] can help in selecting a set of predicted high affinity/selective ligands for ATP12A to be tested in in vitro assays for validating and quantifying in vitro binding assays and their affinity for ATP12A, calculating their kinetic parameters and at the same time verifying their selectivity towards ATP12A.

## 4. Materials and Methods

### 4.1. Semen Samples and Ethics Statement

Frozen/thawed bovine (*Bos taurus taurus,* simply *Bos taurus* in what follows), bubaline (*Bubalus bubalis*) and ovine (*Ovis aries*) semen (*n* = 2 for each species) purchased from a commercial provider (“Centro Tori Chiacchierini” in Perugia, Italy) were used in Western blot and immunofluorescence experiments. Immunofluorescence analysis was also performed on fresh bovine spermatozoa recovered from the testis and different sections of epididymis (*caput*, *corpus* and *cauda*) purchased at a local slaughterhouse (Noicattaro, Bari, Italy) from two adult bulls. As such, no specific approval for this study was required at the Animal Research Ethical Committee (‘Comitato Etico per la sperimentazione animale’, CESA) of University of Bari.

### 4.2. Swim-Up

Frozen sperms were thawed in a humidified incubator and motile spermatozoa were then separated from non-motile ones and a cryoprotective medium by using the swim-up technique. First of all, a modified bicarbonate-free Sperm Analysis Medium [76] containing 150 mM NaCl, 5 mM KCl, 2 mM CaCl_2_, 1 mM MgCl_2_, 10 mM Glucose, 10 mM HEPES and pH 7.4 with NaOH, was prepared. Afterward, 100 µL of semen was incubated in 1 mL of the above-described medium at 37 °C for 1 h in a humidified incubator. After incubation, motile spermatozoa in the supernatant were collected and used for subsequent analyses.

### 4.3. Recovery of Spermatozoa from Testis and Epididymis

The modified bicarbonate-free Sperm Analysis Medium was inoculated with a syringe (needle diameter of 0.30 × 8 mm) into different sections of deferent ducts, tail, body and head of the epididymis and testis after manual slicing of each segment in order to pick up sperm cells.

### 4.4. Polyacrylamide Gel Electrophoresis and Immunoblot Analysis

Protein extracts prepared from frozen/thawed spermatozoa following Muzzachi et al. [77] were used for SDS-PAGE and immunoblotting. In particular, spermatozoa were centrifuged at 16,000× *g* for 5 min, homogenized in lysis buffer (110 mM NaCl, 50 mM TRIS, 0.5% (*v/v*) Triton X-100, 0.5% (*v/v*) Igepal CA-630, pH 8 added with 0.2% (*v/v*) protease inhibitor mixture (Merck/MilliporeSigma, Burlington, MA, USA, P8340), sonicated for 10 s and placed overnight at −80 °C. On the day after, samples were heated at 37 °C for 30 min and centrifuged at 16,000× *g* for 10 min. Supernatant protein concentration was measured by the method of Bradford at 595 nm. Sperm proteins aliquots (30 μg) were diluted in Laemmli buffer, heated at 100 °C for 5 min and separated by 7% (*v/v*) SDS/PAGE gel electrophoresis under reducing conditions. After separation by SDS–PAGE, proteins were transferred onto PVDF membranes (Immobilon P, Merck/MilliporeSigma, Burlington, MA, USA) for immunoblotting. ATP12A was identified by using a polyclonal anti-ATP12A antibody (1:1000 dilution) and targeting the residues 171-306 of ATP12A (A62134, Epigentek, Farmingdale, NY, USA). After washing, blots were incubated with anti-rabbit secondary antibodies (Merck/MilliporeSigma, Burlington, MA, USA) conjugated to peroxidase (1:2000 dilution). Immunocomplexes were detected with the ECL plus reagent (GE Healthcare Life Sciences, Chicago, IL, USA).

### 4.5. Immunofluorescence Analysis

Sperm suspensions were seeded on glass coverslips (25.4 × 76.2 mm), fixed in 4% (*v/v*) paraformaldehyde for 20 min, permeabilized using 0.1% (*v/v*) Triton X-100 in PBS for 10 min and blocked using 0.1% (*w/v*) gelatin in PBS for 10 min at room temperature. Afterward, spermatozoa were incubated for 1 h with different primary antibodies: the polyclonal anti-ATP12A antibody (1:100 dilution) previously adopted in the Western blot analysis; the mouse monoclonal anti-ATP1A1 antibody (1:100 dilution), which recognizes an epitope between amino acid residues 496-506 of the Na^+^/K^+^ ATPase α_1_ subunit (MA3929, Thermo Fisher Scientific, Waltham, MA, USA); the anti-ATP1A4 polyclonal antibody (1:100 dilution), which recognizes the residues 1-90 of the N-terminal region of the α_4_ subunit isoform of Na^+^/K^+^ ATPase (E-AB-65236, Elabscience, Houston, TX, USA); and the anti-ATP1B1 polyclonal antibody (1:100 dilution), which recognizes an epitope between amino acid residues 64-241 of the Na^+^/K^+^ ATPase β_1_ subunit isoform (CSB-PA002326LA01HU, CUSABIO, Houston, TX, USA). Spermatozoa were subsequently incubated with anti-rabbit or anti-mouse IgG secondary antibodies (Merck/MilliporeSigma, Burlington, MA, USA) conjugated to Alexa Fluor 488 or Alexa Fluor 568 (1:1000 dilution) for 1 h. For negative controls, sperms were incubated with only anti-rabbit or anti-mouse IgG secondary antibodies (Merck/MilliporeSigma, Burlington, MA, USA) conjugated to Alexa Fluor 488 or Alexa Fluor 568 (1:1000 dilution) for 1 h.

Coverslips were then mounted onto slides using ProLong Gold Antifade Reagent with DAPI (Thermo Fisher Scientific, Waltham, MA, USA) and examined with NIKON Eclipse 6004 microscope by using 10× or 40× objectives, and images were acquired by NIKON digital camera DXM1200. For acrosomal co-staining [78,79], cells were incubated with TRITC-coupled peanut agglutinin (PNA) (Merck/MilliporeSigma, Burlington, MA, USA) diluted in PBS (final concentration 30 mg/mL) for 30 min at room temperature after secondary antibody incubation. After removing an excess of fluorescence-conjugated lectin by three washes with PBS, the coverslips were processed as mentioned above.

### 4.6. Protein Sequence Sampling and Multiple Sequence Alignment (MSA)

Sequences homologous to the α subunit of non-gastric H^+^/K^+^ ATPase (ATP12A) were collected from the *RefSeq* protein database (https://www.ncbi.nlm.nih.gov/refseq/, accessed on 5 November 2021) and from the *nr* protein database (https://www.ncbi.nlm.nih.gov/refseq/about/nonredundantproteins/, accessed on 5 November 2021) using *blastp* searches (with default parameters). The *B. taurus* ATP12A sequence (XP_002691916.3) was used as a query sequence to search for homologous sequences in *Mammalia* (taxid:40674), *Aves* (taxid:8782), *Amphibia* (taxid:8292), *Lepidosauria* (taxid:8504), *Arthropoda* (taxid:6656), *Chondrichthyes* (taxid:7777), *Nematoda* (taxid:6231), *Fungi* (taxid:4751), *Embryophyta* (taxid:3193) and *Bacteria* (taxid:2) according to protocols previously described [80,81,82,83,84]. The 100 best protein hits, highlighted by using *blastp* searches, were retrieved for each taxonomic group. Among the sampled sequences, five protein sequences for each taxonomic group were selected for subsequent Multiple Sequence Alignment (MSA) analysis. Truncated sequences were excluded from the following comparative analysis. All selected ATP12A homologous sequences (36 from metazoan, 7 from fungi, 3 from plants and 6 from bacteria) showed E-values equal to zero, query coverage greater than 75% and percentage of identical residues greater than 60% in the performed *blastp* searches.

Given that ATP12A homologous sequences were detected in all cited taxonomic groups and due to the high amino acid conservation observed among H^+^/K^+^ ATPase and Na^+^/K^+^ ATPase family members [11,85], ATP4A (14 sequences), ATP1A1 (19 sequences), ATP1A2 (10 sequences), ATP1A3 (10 sequences) and ATP1A4 (9 sequences) were sampled from *Mammalia* for comparative purposes. Sequences homologous from *B. taurus* and *H. sapiens* to ATP1B1, ATP1B2, ATP1B3, ATP1B4 and ATP4B were also sampled from *Mammalia* (taxid:40674) using the same approach described above and used as outlier sequences for subsequent phylogenetic analyses and comparative and structural analyses aiming to predict a putative β subunit interactor of ATP12A. An MSA of the 122 sampled α and β subunits of the considered P2C-ATPases fulfilling the parameters described above was built by using *ClustalW* implemented in the sequence editor package *Jalview* [86]. No redundancy was observed in the obtained MSA.

### 4.7. Phylogenetic Analysis

The analysis of evolutionary relationships among homologous ATP12A, ATP1A1-4, ATP4A sequences sampled by *blastp* was conducted using *MEGA11* [87] starting from the MSA of the above cited 122 homologous protein sequences. As mentioned in the previous paragraph, ATP1B1-4 and ATP4B mammalian sequences were used as outliers. In detail, the tree was built from the ungaped MSA by applying the maximum likelihood method with the *JTT* model for amino acid substitutions and a gamma distribution (five discrete gamma categories) for the rates among sites. A total of 100 bootstrap samplings were applied to test the robustness of the tree, as previously described in [80,81,82,83,84].

### 4.8. 3D Comparative Modeling of Bovine ATP12A, Bovine ATP4B, Bovine ATP12A.ATP1B1 and Bovine ATP12A.ATP4B Protein Complexes

Since 2007, several crystallized structures of ATP1A1 (i.e., 3b8e.pdb and 4xe5.pdb) [88] and ATP4A (5ylu.pdb, 5ksd.pdb) [89] have been solved. For verifying the presence of recent crystallized structures for the other P2C-ATPases, we screened the protein data bank by using the fold recognition tools of pGenTHREADER and i-Tasser. Notably, both i-Tasser and pGenThreader confirmed that other P2C-ATPases have not been crystallized yet and suggested *Sus scrofa* ATP4A (5ylu.pdb) and *Bos taurus* ATP1A1 (4xe5.pdb) as good protein templates for guiding 3D protein modeling of ATP12A and ATP1A4. The 3D comparative models of *B. taurus* ATP12A were built by using *B.taurus* ATP1A1 and *S. scrofa* ATP4A as protein templates, as suggested by I-TASSER and pGenThreader.

The 3D comparative model of ATP12A was obtained by multi-template comparative modeling by using crystallized bovine ATP1A1 (chain A of 4xe5.pdb) [88] and crystallized swine ATP4A (chain A of 5ylu.pdb) [89]. The 3D comparative model of bovine ATP4B was built by using the crystallized ATP4B from *Sus scrofa* (chain B of 5ylu.pdb) as a protein template. The 3D comparative model of bovine ATP12A.ATP1B1 was obtained by superimposing the 3D comparative model of ATP12A on the ATP1A1 chain of the crystallized bovine ATP1A1.ATP1B1 protein complex (4xe5.pdb) accordingly to protocols previously described (see [80,81,82,83,84]). Coordinates of the resulting ATP12A.ATP1B1 bovine protein complex were minimized by using the *yasara* minimization server. The 3D comparative model of bovine ATP12A.ATP4B was obtained by superimposing the 3D comparative models of ATP12A and ATP4B on the ATP4A chain and the ATP4B chain, respectively, of the crystallized swine ATP4A.ATP4B protein complex (5ylu.pdb) [89] (for reference, see [80,81,82,83,84]). Again, coordinates of the resulting ATP12A.ATP4B bovine protein complex were minimized by using the *yasara* minimization server [90].

### 4.9. Interaction Energy Calculations

The FoldX AnalyseComplex assay [91] was performed to determine the interaction energy between the ATP1A1 and ATP1B1 protein subunits within the crystallized bovine ATP1A1.ATP1B1 protein complex and between the ATP4A and ATP4B protein subunits within the crystallized swine ATP4A.ATP4B protein complex for comparative purposes. The same assay was performed to determine the interaction energy between the ATP12A and ATP1B1 protein subunits within the modelled bovine ATP12A.ATP1B1 protein complex. The interaction energy between the ATP12A and ATP4B protein subunits within the modelled bovine ATP12A.ATP4B protein complex was calculated for comparative purposes. More negative energies indicate better binding. Positive energies indicate no binding [91,92].

## Figures and Tables

**Table 1:** List of X-K-ATPases transporters.

Protein Complex	Subunit α	Subunit β
Na^+^/K^+^ ATPase	α_1_ (ATP1A1)	β_1_ (ATP1B1)
	α_2_ (ATP1A2)	β_2_ (ATP1B2)
	α_3_ (ATP1A3)	β_3_ (ATP1B3)
	α_4_ (ATP1A4)	β_4_ (ATP1B4)
“gastric” H^+^/K^+^ ATPase	α (ATP4A)	β (ATP4B)
“non gastric” H^+^/K^+^ ATPase	α (ATP12A)	β non-specific

## Supplementary Materials

The following supporting information can be downloaded at: https://www.mdpi.com/article/10.3390/ijms23031048/s1.
