# Supplementary material for: The Non-Gastric H+/K+ ATPase (ATP12A) Is Expressed in Mammalian Spermatozoa
Source: Int J Mol Sci. 2022 Jan 19;23(3):1048. doi: 10.3390/ijms23031048 (PMC8835340; doi:10.3390/ijms23031048)
Supplement: Supplementary file 1 [file ijms-23-01048-s001.zip › ijms-1474262-supplementary.pdf]

The Non-Gastric H^+/K^+ ATPase (ATP12A) Is Expressed in Mammalian Spermatozoa

Supplementary Material

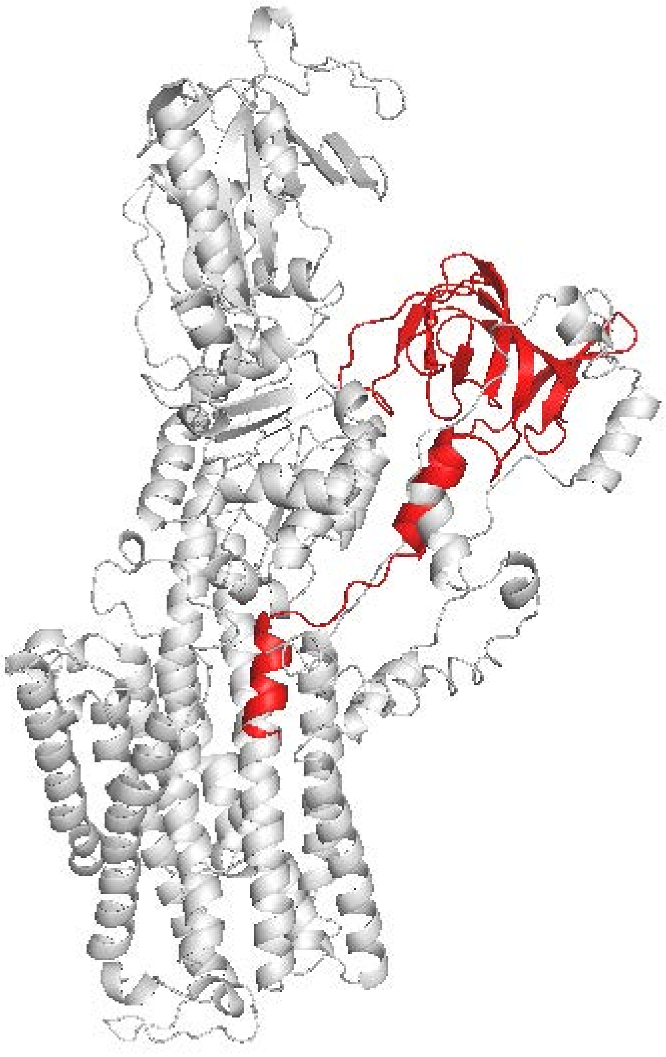

Figure S1. The 3D comparative model of bovine ATP12A is reported in white cartoon representation. Red cartoon indicates the localization of residues 171-306, which form the target region of the employed A62134 EpiGentek antibody.

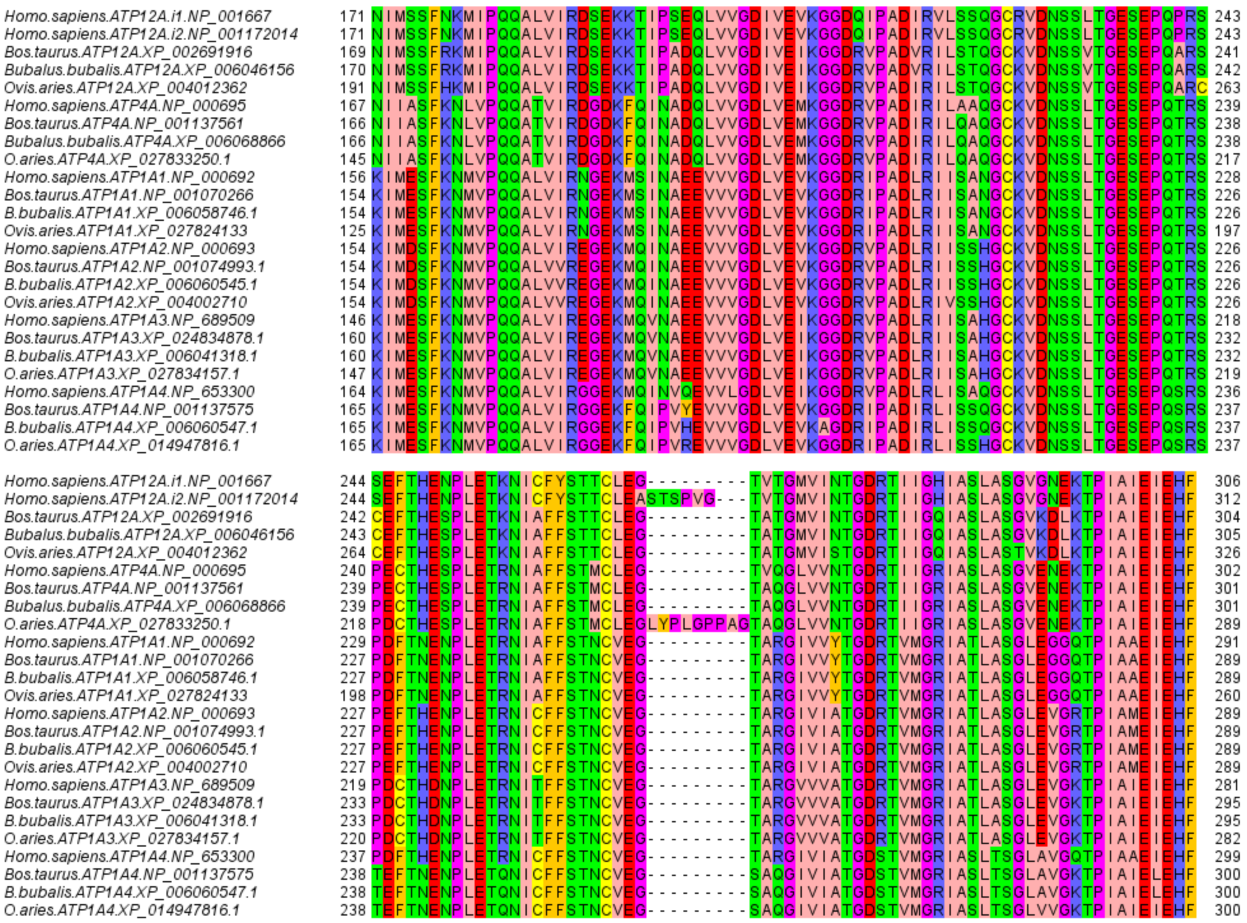

Figure S2. MSA of residues from the different sampled ATP1A1-4, ATP12A and ATP4A sequences, aligned with 171-306 residues from the bovine ATP12A, forming the target region of the employed A62134 EpiGentek antibody.

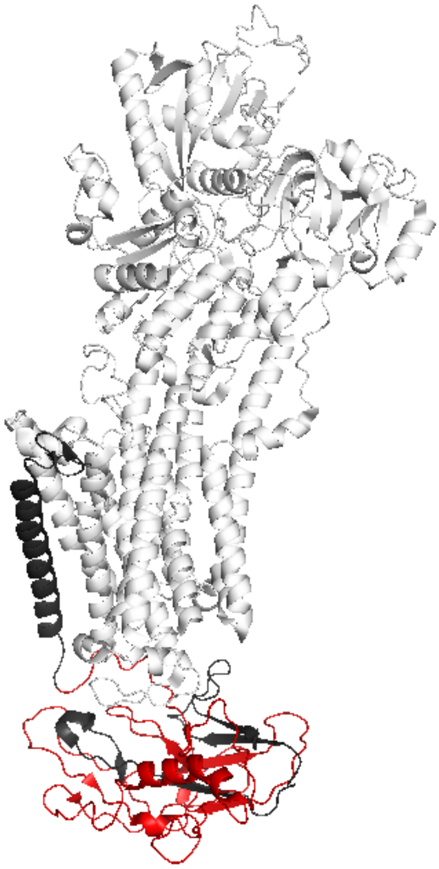

Figure S3. The 3D crystallized structure of bovine ATP1A1 (white cartoon representation) in complex with ATP1B1 (black cartoon representation) is reported. Red cartoon indicates the localization of residues 64-241, which form the target region of the employed CSB-PA002326LA01HU antibody.

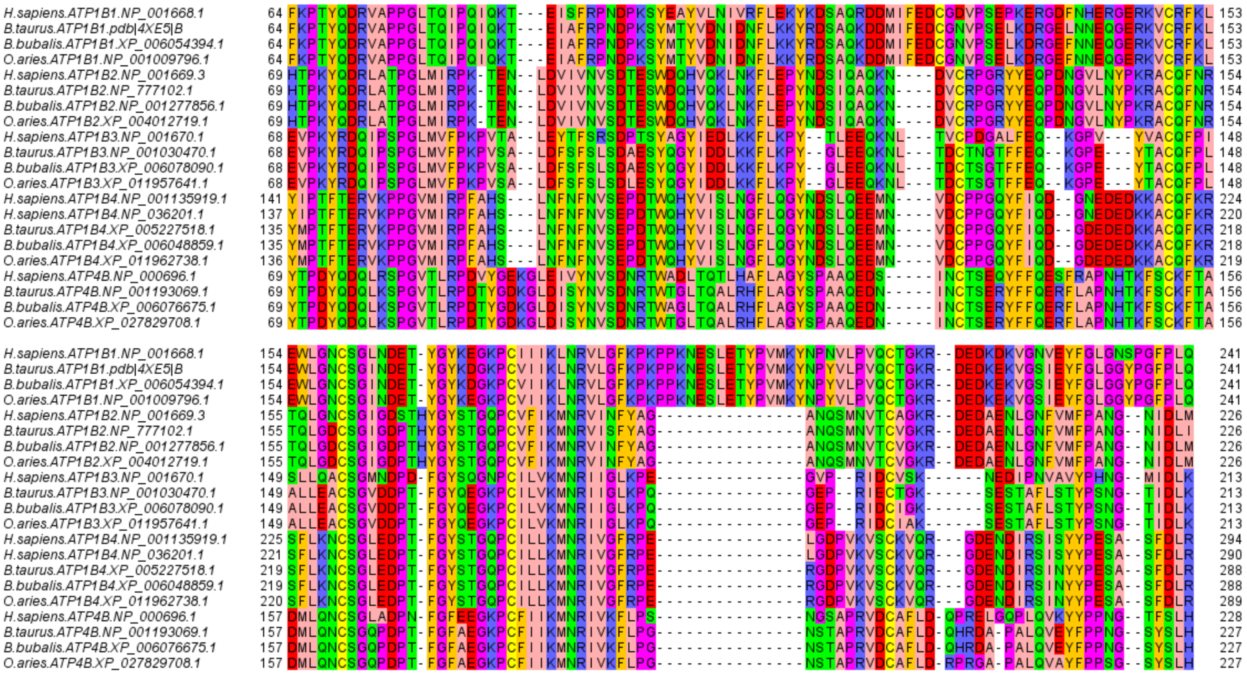

Figure S4. MSA of residues from the different sampled ATP1B1-4 and ATP4B sequences, aligned with 64-241 residues from the bovine ATP1B1, forming the target region of the employed CSB-PA002326LA01HU antibody.

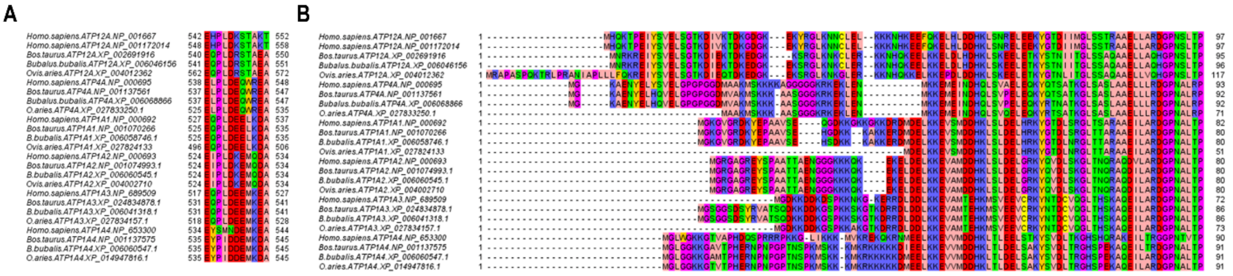

Figure S5. (**A**) MSA of residues from the different sampled ATP1A1-4, ATP12A and ATP4A sequences, aligned with 496-506 residues from lamb ATP1A1, forming the target region of the employed MA3-929 Invitrogen antibody. (**B**) MSA of residues from the different sampled ATP1A1-4, ATP12A and ATP4A sequences, aligned with 496-506 residues from the lamb ATP1A1, forming the target region of the employed MA3-929 Invitrogen antibody.

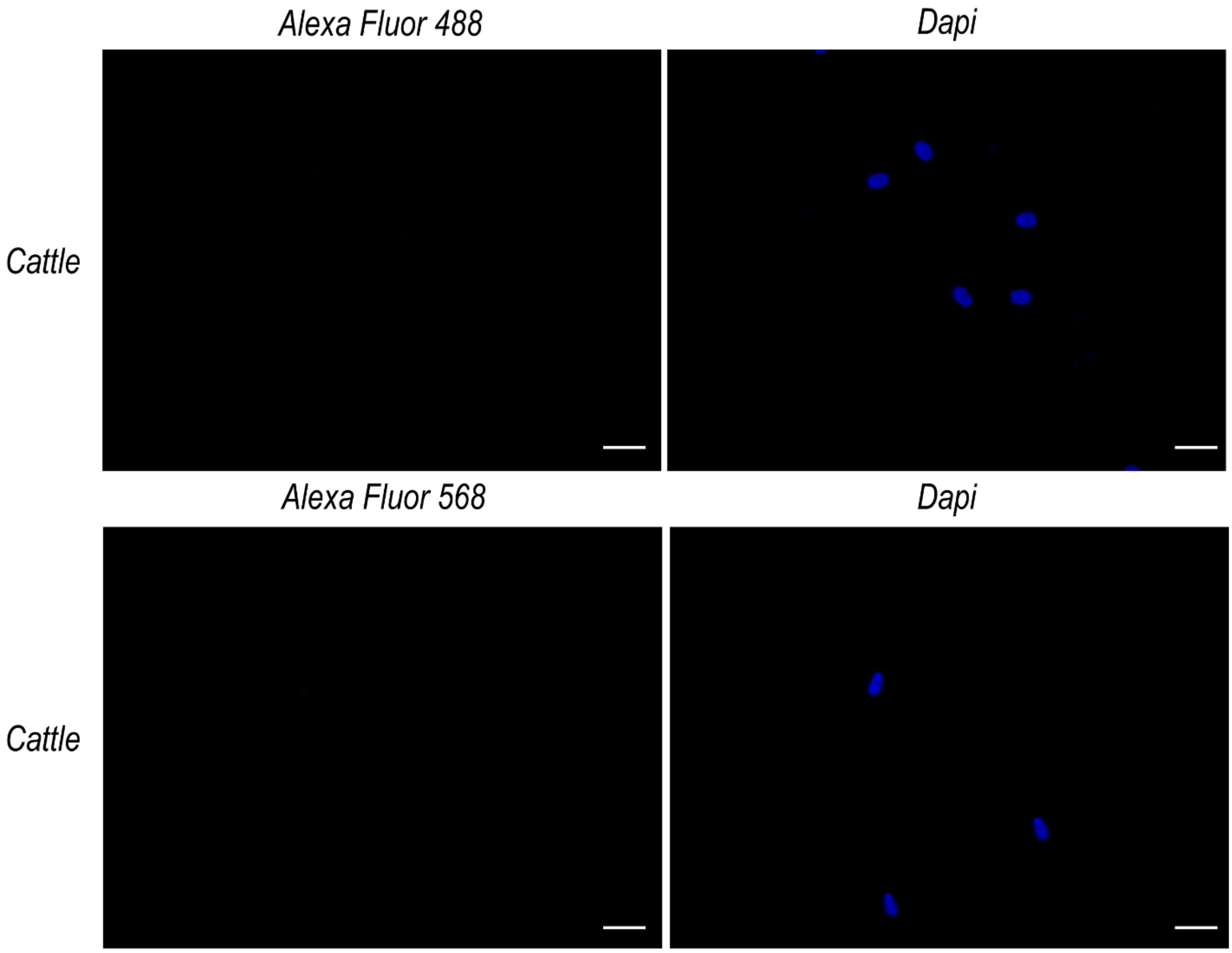

Figure S6. Negative controls sperms were incubated with either anti-rabbit or anti-mouse IgG secondary antibodies conjugated to Alexa Fluor 488 (upper panels) or Alexa Fluor 568 (lower panels), respectively. Nuclei were counterstained with DAPI (blue). Scale bar: 20 μm.
